# Supplementary material for: Developing an AI-assisted clinical decision support system to enhance in-patient holistic health care
Source: PLoS One. 2022 Oct 31;17(10):e0276501. doi: 10.1371/journal.pone.0276501 (PMC9621444; doi:10.1371/journal.pone.0276501)
Supplement: Attachment — Submitted filename: Plos review comment+response 20220717.docx [file pone.0276501.s001.docx]

**Paper No.**: Plos One [PONE-D-21-31708]

**Title**: Developing an AI-assisted Clinical Decision Support System to Enhance In-patient Holistic Health Care
**Authors**: Ming-Hsia Hsu, Wang-Chuan Juang, Zheng-Xun Cai, Li-Chu Wu,

Wei-Chun Huan,Tsuang Kuo, and Chia-Mei Chen

# Comments & Responses:

**Reviewer #1:**

The manuscript is of a great effort, particularly collecting patients heath data. However, it will be wonderful to address some pints mentioned below:

| Comment | Response |
| --- | --- |
| 1. Abstract should be written in one or two paragraphs without the subsections. One is most preferred. It is the summary of entire manuscript. | The abstract has been revised accordingly.  The revised portion is marked in navy blue color. |
| 2. There is no much contribution on proposed method. If the authors does not brings novelty in the method, at least make the comparison with at least four to five existing methods/algorithms. | Thank you for pointing out the confusion.  The novelty is explained in Discussion, followed by its strength. The revised portion is marked in navy blue color.  Based on our best knowledge, this is the first attempt to apply time-series machine learning model to predict patient’s medical need. |
| 3. The Performance matrix provided in the result section is not enough. The readers would like to see more analysis, like precision, recall, f1 measure etc. | Thank you for pointing out the confusion.  To improve the readability, the revision splits Table 6 which contains the confusion matrix and performance metrics into two tables: one for the confusion matrix and the other for all the performance metrics including the suggested precision, recall, and F1 measures.  The revised portion is marked in navy blue color. |
| 4. The references are not enough. adding more state-of-the-art references would bring value to the manuscript. | Thank you for the valuable suggestion.  The revision has reviewed extra 9 pieces of contemporary related research to enhance the value of this work. The added references are marked in navy blue color. |

**Reviewer #2:**

It is very interesting to read your work on Developing an AI-assisted Clinical Decision Support System to Enhance In-patient Holistic Health Care. I found the study contributing to the body of knowledge. It wIll be interesting to read your further works.

| Comment | Response |
| --- | --- |
| It is very interesting to read your work on Developing an AI-assisted Clinical Decision Support System to Enhance In-patient Holistic Health Care. I found the study contributing to the body of knowledge. It wIll be interesting to read your further works. | Thank you for your comment. We will be honored to have our work published in the journal. |
